# 2447 Community voices first: A multi-method approach to shaping institutional response to Flint’s water crisis

**DOI:** 10.1017/cts.2018.243

**Published:** 2018-11-21

**Authors:** Karen D. Calhoun, Kent Key, E. Yvonne Lewis, Jennifer Carerra, Joseph Hamm, Susan Woolford, E. Hill De Loney, Ella Greene-Moten, Arlene Sparks, Don Vereen, Patricia Piechowski-Whitney, Kaneesha Wallace, Ismael Byers, Athena McKay, DeWaun Robinson, Jess Holzer, Vanessa De Danzine, Adam Paberzs, Meghan Spiroff, Erica Marsh

**Affiliations:** 1 University of Michigan School of Medicine, Ann Arbor, MI, USA; 2 Dare2Dream, Flint, MI, USA; 3 University of Michigan School of Public Health, Ann Arbor, MI, USA; 4 Institute for Clinical and Health Research, University of Michigan, Ann Arbor, MI, USA; 5 Healthy Flint Research Coordinating Center, Flint, MI, USA; 6 Hope College, Holland, MI, USA; 7 Artistic Vision Enterprise, Flint, MI, USA; 8 Hofstra University, Long Island, NY, USA; 9 New York City Police Department, New York City, NY, USA; 10 University of Michigan Health System and Hospitals, Ann Arbor, MI, USA

## Abstract

OBJECTIVES/SPECIFIC AIMS: Explore perceptions of Flint stakeholders on the water crisis regarding trust and the capacity of faith and community-based organizations providing public health services to address community needs. Analyze the community’s voice shared at (1) 17 key community communications (community/congressional meetings and events), and (2) during 9 focus group sessions, in which residents, faith-based leadership and other stakeholders discuss issues and concerns on the Flint Water Crisis, and recommend ways to address them. Develop a framework that defines core theories, concepts and strategies recommended by the community to help rebuild trust and the quality of life in Flint, Michigan, and support other communities experiencing environmental stress. METHODS/STUDY POPULATION: Study population: faith-based leaders, seniors, youth, Hispanic/Latino and African American stakeholders, and others experiencing inequities in the city of Flint. Convene 9 focus group sessions (recorded and transcribed) to learn community perceptions on trust and ways to address it. Validate accuracy of the transcriptions with community consultants to reconcile any inaccurate information. Through a community engaged research (CEnR) process, review and analyze qualitative data from the 9 focus group sessions, and quantitative data from 2 surveys documenting (1) demographic backgrounds of focus group participants, and (2) their perceptions on trust and mistrust. Prepare a codebook to qualitatively analyze the focus group data summarizing community input on trust, mistrust, changes in service delivery among community and faith-based organizations, and ways to re-build trust in the city of Flint. Transcribe the community’s voice shared during 17 key events, identified by a team of community-academic stakeholders (i.e., UM Flint water course, congressional and community events, etc.), in which residents and other stakeholders discuss issues and concerns on the Flint Water Crisis, and recommend ways to address it. Qualitatively analyze the transcriptions, using a CEnR process to prepare a codebook on key themes from the community’s voice shared at these events, and recommendations on ways to address it. Compare and contrast findings between the two codebooks developed from (1) the focus group data and (2) qualitative analysis of community voice during public meetings and events. Synthesize this information into a framework of core theories, concepts and rebuilding strategies for Flint, Michigan. RESULTS/ANTICIPATED RESULTS: It is important to note many undocumented immigrant populations in Flint fear deportation and other consequences, hampering their ability to obtain service and provide community voice. Through our purposive sampling approach, we will hear from community voices not often included in narratives (i.e., seniors, youth, Hispanic/Latino residents). The presentation will present findings documenting levels of trust and mistrust in the city of Flint; and a framework of recommendations, core theories and concepts on ways to reduce, rebuild and eliminate stress that will be helpful to other communities experiencing distress. DISCUSSION/SIGNIFICANCE OF IMPACT: To our knowledge, levels of trust and mistrust in Flint have not been documented thus far. We will compare and contrast common themes presented by the community at public meetings and events with themes presented in our focus group effort on trust. Faith and community-based providers were among the first responders to the Flint Water Crisis. The effort will also share perceptions on changes in public health service delivery, and observations on preparedness for these roles that occurred among community and faith-based providers. Finally, the effort will (1) support the design of a research agenda, (2) define a framework of core theories, concepts and recommendations developed by the community to help rebuild trust in Flint, Michigan; and (3) support other communities addressing environmental distress.

